# Exploring the Link between Uric Acid and Osteoarthritis

**DOI:** 10.3389/fmed.2017.00225

**Published:** 2017-12-13

**Authors:** Cheryl Ann Ma, Ying Ying Leung

**Affiliations:** ^1^Duke-NUS Medical School, Singapore, Singapore; ^2^Department of Rheumatology and Immunology, Singapore General Hospital, Singapore, Singapore

**Keywords:** Gout, hyperuricemia, osteoarthritis, epidemiologic study, inflammation, therapeutic use

## Abstract

Both gout and osteoarthritis (OA) are common forms of arthritis that inflict a huge burden to an aging population with the increasing prevalence of obesity. Clinicians have long observed the link between these two conditions. In this review, we summarize the evidence from epidemiologic and immunological studies that described the possible relationship between the two conditions. The recent new understanding on monosodium uric acid crystal-induced inflammation has given insight into probable shared pathogenesis pathways for both conditions. We describe the potential therapeutic implications, particularly regarding the possibility of repurposing traditional gout medications for use in OA.

## Introduction

The risk of mobility and disability attributable to osteoarthritis (OA) alone is greater than any other medical condition among elderly (1). The prevalence of OA that are symptomatic varies between 7 and 26% dependent on the site and definition of OA (2). The burden of OA has been increasing in the past two decades worldwide (3), and its prevalence is projected to be nearly double in the next decade (4). Current treatment paradigms are limited to palliative measures broadly focused on analgesia and joint replacement for end stage disease. Gout is a crystal-induced arthritis caused by deposition of the monosodium uric acid crystal (MSU) related to long standing hyperuricemia. It is also a common inflammatory arthritis affecting around 5% of the middle-aged and elderly population worldwide (5). The association of uric acid and OA has long been observed, and a pathological link between gout and OA has been hypothesized (6). Aging and obesity are significant risk factors shared by both OA (7, 8) and gout (9), and may confound the association between both conditions. The pathological links between these two common conditions remain elusive. Whether gout promotes the development or progression of OA is particularly relevant to OA research. Thus far no drug has been approved for structural protection of the joint or prolongation of joint life in OA despite the high prevalence, individual impact of disability, and high societal cost of OA. Therefore should gout and OA prove to have a causative relationship, and share common pathological pathway, effective treatment for gout might potentially be useful for OA. In this narrative review, the possible associations and pathological mechanisms between hyperuricemia, gout, and OA are discussed.

## Methods

**Figure 1:**
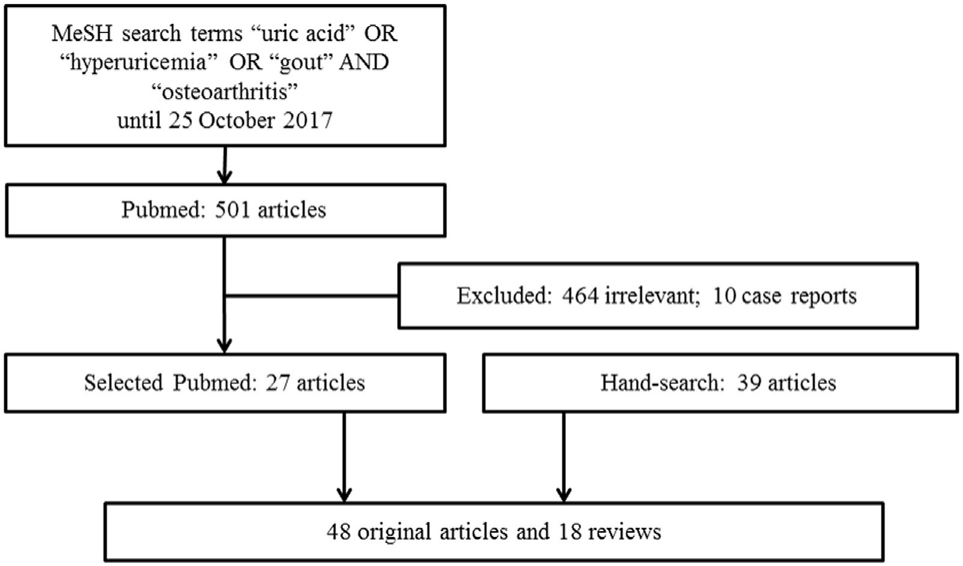
Flowchart on Article Selection.

We conducted a literature search from the PubMed database for relevant studies published using the MeSH search terms “uric acid” OR “hyperuricemia” OR “gout” AND “osteoarthritis” until 25 October 2017. The search was restricted to articles written in English. Articles that provided information of a link between hyperuricemia or gout with OA were included. We excluded case reports or small case series. Additionally, we hand searched and reviewed references from the relevant articles. A total of 501 articles published in English were identified from the PubMed search and 464 of these articles were deemed to be outside the scope of this review and 10 case reports were excluded. A total of 27 relevant articles from the search, 39 articles from relevant references from the hand search and other resources were retrieved for full text review. The final review included 66 articles, with 48 original articles and 18 reviews (Figure 1). Despite a preliminary search, this review is narrative in nature rather than systematic and therefore represents the authors’ current understanding of the topic.

## Results

### Evidence of Associations from Epidemiologic Studies

Subjects with OA in various sites had higher serum uric acid level compared to healthy controls. From a population-based cross-sectional study, independent associations between serum uric acid concentration with hand OA, and OA of other body sites were revealed (10). Several subsequent population-based cross-sectional studies (11, 12) and cohort studies (13–15) have noted positive associations of uric acid concentration with OA, but these associations became insignificant when body mass index (BMI) was controlled as confounder. On the other hand, uric acid levels at the highest tertile may be associated with OA. In a cross-sectional study, the highest serum uric acid tertile were associated with generalized OA in subjects who had undergone arthroplasty for hip OA, but not with those who had undergone arthroplasty for knee OA (16). Another cross-sectional study involving over 4,000 participants in China has shown that women with the highest tertile of uric acid showed the highest degree of radiographic features suggestive of knee OA after adjusted for various confounding risk factors including BMI (17). Recently, a study of knee OA subjects without gout showed that serum uric acid levels significantly distinguished non-progressors from progressors defined by joint space narrowing on radiography over 24 months (18). We summarized the evidence from epidemiologic studies in Table 1.

**Table 1:** Summary of epidemiologic evidence of hyperuricemia or gout with OA.

Reference	Study name	Country	Design	Sample size	Study variable	Site of OA	Definition of OA	Main outcomes	Adjustment
**Hyperuricemia**									
Acheson et al. (10)	New Haven 1960 Census	USA	Cross-sectional	Cases: 685 Control: 1,704	UA	Hand, whole body	Radio-graphic	– UA was associated with hand OA and body site OA among women but not men (univariate analysis). – UA was associated with whole body OA in men but not women (adjusted).	Age, gender, weight/height

Anderson et al. (11)	Health and Nutrition Examination Survey	USA	Cross-sectional	Cases: 315 Control: 4,878	UA	Knee	Clinical and radio-graphic	– UA was associated with increase in risk of knee OA in women (age adjusted OR 1.27; 95% CI 1.15–1.40). – Association was not significant after adjustment of BMI.	Age, BMI, and other variables

Hart et al. (12)	Chingford Study	UK	Cross-sectional	Cases: 118 Control: 861	UA	Knee	Radio-graphic	– UA was not associated with knee OA after adjustment for age and BMI.	Age, BMI

Sun et al. (16)	Ulm Osteoarthritis Study	Germany	Cross-sectional	809	UA	Knee, hip, whole body	Radio-graphic	– Highest tertile of UA was associated with generalized OA in subjects with previous hip arthroplasty for OA (adjusted OR 3.5; 95% CI 1.3–9.1) but not in knee arthroplasty.	Age, gender, BMI, diuretics use, and other variables

Ding et al. (17)	–	China	Cross-sectional	4,685	UA	Knee	Radio-graphic	– Highest tertile of UA was associated with OST in women (adjusted OR 1.43; 95% CI 1.01–2.03). – No association between UA and OST in men. – No association between UA and JSN was observed in both men and women.	Age, BMI, and other factors

Felson et al. (13)	Framingham Heart Study Cohort	USA	Cohort	1,420	UA	Knee	Clinical and radio-graphic	– UA was not associated with knee OA after adjustment of BMI and other factors in both genders.	Age, BMI, physical activity

Bagge et al. (14)	–	Sweden	Cohort	538	UA	Knee	Radio-graphic	– UA was associated with knee OA in women (*P* < 0.01) but not in men. – Association was not significant after adjustment of BMI.	BMI

Schouten et al. (15)	–	Netherlands	Cohort	142	UA	Knee	Radio-graphic	– Highest tertile of UA was associated with loss of joint space width (OR 1.36; 95% CI 0.46–4.02). – Association was not significant after adjustment for age, gender and BMI.	Age, gender, BMI

Krasnokutsky et al. (18)		USA	Cohort	88	UA	Knee	Clinical and radio-graphic	– UA was associated with JSN. – UA distinguished progressors (JSN > 0.2 mm) and fast progressors (JSN > 0.5 mm) from non-progressors (JSN ≤ 0.0 mm) [AUC 0.63, *P* = 0.03; and 0.62, *P* = 0.05, respectively]. – Association was significant after adjustment for age, gender and BMI.	Age, gender, BMI

**Gout**									
Roddy et al. (21)	–	UK	Cross-sectional	Patients: 164 Joint sites: 5,904	Gout	Hand, knee, hip	Previous TKR or clinical	– Site of gout attacks was associated with the presence of OA (adjusted OR 7.94; 95% CI 6.27–10.05). – Associations between acute gout attacks and presence of OA: 1st MTP joint: adjusted OR 2.06; 95% CI 1.28–3.30 Mid-foot: adjusted OR 2.85; 95% CI 1.34–6.03 Knee: adjusted OR 3.07; 95% CI 1.05–8.96 DIP joints: adjusted OR 12.67; 95% CI 1.46–109.9	Age, gender, BMI, diuretics use

Bevis et al. (23)	–	UK	Cross-sectional	Case: 53 Controls: 221	Gout	Hand, knee, foot	Radio-graphic	– No associations were observed between gout and radiographic hand, knee or foot OA. – Gout had odds of having: Nodal hand OA (adjusted OR 1.46; 95% CI 0.61–3.50) Foot OA (adjusted OR 2.16; 95% CI 0.66–7.06) Knee OA (adjusted OR 0.57; 95% CI 0.20–1.65)	BMI, diuretic use, and other factors

Howard et al. (24)	–	USA	Cross-sectional	Gout: 25 UA: 25 Health control: 25	Gout	Knee	Clinical and radio-graphic (ACR criteria)	– 68.0% of gout, 52.0% of asymptomatic hyperuricemia, and 28.0% of age-matched control subjects had knee OA (gout vs. control, *P* = 0.017). – Gout was associated with knee OA (OR 5.46; 95% 1.63, 18.36. *P* = 0.006). Risk reduced after adjustment with BMI (OR 3.80, 1.60, 13.57. *P* = 0.040) – HA was not significantly associated with knee OA – Knee OA was more severe in gout patients vs. controls (mean KL grade: 3.50 vs. 2.38, *P* = 0.001).	BMI

Lally et al. (19)	70-year-old people in Göteborg	USA	Case–control	149	Gout	Hand	Radio-graphic	– 17% of gout patients had nodal hand OA. – 80% of OA patient had radiographic criteria for gout around the IP joints.	No adjustment

Fam et al. (20)	–	Canada	Case–control	32	Gout	Hand	Physician diagnosis	– In 32 subjects with nodal hand OA, 90% have gouty tophi in the PIP joints and DIP joints.	No adjustment

Roddy et al. (22)	–	UK	Case–control	Cases: 164 Controls: 656	Gout	Hand, Knee, Toe	Self-reported	– Gout was associated with knee pain (adjusted OR 2.05; 95% CI 1.37–3.06), hallux valgus (adjusted OR 2.10; 95% CI 1.39–3.18) and big toe pain (adjusted OR 2.94; 95% CI 1.62–5.34).	BMI, diuretic use

Kuo et al. (26)	–	UK	Case–control	Case: 39,111 Controls: 39,111	Gout	All	Physician diagnosis (database)	– OA diagnosis 10 years prior to incident gout is associated gout (OR 1.27) – Gout was significantly associated with a 1-, 2-, 5-, and 10-year risk of OA (adjusted OR 1.45; 95% CI 1.35–1.54)	Age, gender, BMI, and other factors

Teng et al. (25)	Singapore Chinese Health Study	Singapore	Cohort	51,858	Gout	Knee	Incident TKR (registry)	– Gout was associated with risk of TKR in women (adjusted HR 1.39; 95% CI 1.08–1.79) but not in men (adjusted HR 0.78; 95% CI 0.49–1.23). – Association was stronger in women who were lean (adjusted HR 2.17; 95% CI 1.30–3.64) compared to heavier counterparts (adjusted HR 1.24; 95% CI 0.93–1.66).	Age, gender, BMI, and other factors

*USA, United States of American; UK, United Kingdom; UA, uric acid levels; OR, odds ratio; HR, hazard ratio; 95% CI, 95% confidence interval; ACR, American College of Rheumatology; OA, osteoarthritis; TKR, total knee replacement; BMI, body mass index; OST, osteophytes; JSN, joint space narrowing; AUC, area under the receiver operating characteristic curve; IP, interphalangeal; PIP, proximal interphalangeal; DIP, distal interphalangeal; MTP, metatarsophalangeal*.

It is important to distinguish between hyperuricemia and gout, where gout is the clinical event resulting from MSU deposition. Co-localization of gout and nodular hand OA has long been recognized (19, 20). In a nested case–control study from adults registered with two general practices in Nottingham, history of gouty attacks in individual joint sites was strongly associated with the presence of clinically assessed OA after adjusting for age, gender, BMI, and prior diuretic use (21). In particular, the first metatarsophalangeal joint, mid-foot, knee, and distal interphalangeal joint of finger that was affected by gouty attacks had a 2.1-fold, 2.9-fold, 3.1-fold, and 12.7-fold increased likelihood of having concurrent OA that was clinically defined. In another study using the same dataset, Roddy et al. reported that joints affected by gouty arthritis were more likely to manifest chronic pain symptoms of OA (22). Subsequently, the same group evaluated the risk of radiographic defined OA in a cross-sectional study nested within three observational clinic cohorts of subjects aged ≥50 years with joint pain. Comparing with 211 age- and gender-matched control without gout, 53 subjects with gout had an increased risk of hand and foot OA; but reduced risk of knee OA (23). Although underpowered, these studies suggest that either joints that have underlying OA may predispose to MSU crystal deposition, or the damage caused by previous gouty arthritis may predispose to OA. Howard et al. assessed for clinical and radiographic knee OA in a small but well-defined cohort of middle-aged to elderly men with hyperuricemia, gout, or neither condition. Participants with gout had an increased prevalence of knee OA compared to participants without gout. The presence of gout was associated with more severe structural knee OA on radiography and more frequent bilateral knee involvement. However, the differences between the hyperuricemia group and the control and/or gout groups did not achieve statistical significance (24).

The cross-sectional design of all the above studies cannot differentiate whether gout predisposes to the development of OA or joints with OA facilitate the localized deposition of MSU crystals in presence of hyperuricemia. Our research group recently reported the first cohort study in self-reported physician diagnosed gout in risk of knee arthroplasty due to severe OA. Among the 51,858 participants and 1,435 cases of incident knee arthroplasty after a mean follow-up of 9.7 years, we demonstrated a significantly increased risk of knee arthroplasty among women with gout (adjusted hazard ratio, HR = 1.39), adjusted for all possible risk variables, including age and BMI (25). Interestingly, this increased risk of arthroplasty was stronger in lean women with gout compared to their heavier counterparts [HR 2.17 (95% CI: 1.30–3.64) vs. 1.24 (95% CI: 0.93–1.66) among women with BMI below or above 23 kg/m^2^]. The stronger association between gout and risk of arthroplasty among lean subjects strengthens the observed association between gout and knee OA, as it is not confounded by BMI. The association in men was not significant, likely related to the small number of knee arthroplasty among men. As there was no information of knee OA at baseline, we were not able to discern whether self-reported gout is associated with the onset or the progression of severe knee OA (25). In a case–control study, Kuo et al. evaluated the comorbidities among 39,111 subjects with incident gout and 39,111 matched controls identified from a retrospective clinical registry data that is representative of the UK population from 1997 to 2005 (26). Subjects who received a diagnosis of OA 10 years prior to the index date of incident gout had a higher risk for incident gout (OR 1.27), after adjustment of multiple variables including age and BMI. Conversely, among participants without OA at baseline, incident gout was significantly associated with increased 1-, 2-, 5-, and 10-year risk of development of OA (overall adjusted HR of 1.45). This study suggested a bi-directional relationship: gout is associated with a higher risk of incident OA, while OA is positively related to incident gout.

### Molecular Evidence for Possible Pathological Link between Gout and OA

Gout may promote cartilage degradation due to the direct effects of MSU crystals. MSU crystals have been shown to inhibit human chondrocyte viability and function *in vitro*, in a dose-dependent manner (27). Similar phenomena were observed in *ex vivo* cartilage explant cultured with MSU crystals, which led to a rapid increase in expression of the degradation enzymes aggrecanase (ADAMTS4 and ADAMTS5), and reduction in formation of the matrix proteins aggrecan, versican, and collagen type 2α1 (27). From a study of 7855 cadaveric talar surfaces of ankles from 4,007 donors, deposits of MSU and calcium pyrophosphate dihydrate (CPPD) crystals were located in regions that were subjected to biomechanically stress or joint instability. Crystal deposits were strongly associated with cartilage degradation, and immunohistological changes of cartilage degradation and repair (28). However, a minority (8%) of talus with crystal deposits showed no evidence of cartilage degeneration; and therefore, cartilage damage may not be a pre-requisite for crystal deposition.

Recent advances in the understanding of the pathophysiology of crystal-induced inflammation have provided insight into a possible shared inflammatory pathway between gout and OA (Figure 1). MSU crystals activate the macrophage innate immune response via the Nacht Domain, leucine-rich repeat, and pyrin domain-containing protein 3 (NALP3) inflammasome, that is required for caspase-1 activation and subsequent interleukin (IL)-1β and IL-18 processing and release (29). The IL-1β family cytokines, particularly IL-1β, have been heavily implicated in the pathogenesis of OA (30). IL-1β is involved in multiple pathways resulting in cartilage destruction – this includes stimulating the production of prostaglandin E2, nitric oxide, cytokines, and chemokines involved in joint inflammation, suppressing type II collagen production, and stimulating the synthesis and activity of matrix metalloproteinase (MMP) and aggrecanase (31). The potential role of IL-1β as a link in pathogenesis for both gout and OA has been demonstrated in the Prediction of Osteoarthritis Progression study (32). In subjects with knee OA without prior clinical history of gout, Denoble et al. showed that the soluble form of uric acid in synovial fluid was strongly associated with synovial fluid IL-18 and IL-1β (32). Furthermore, both synovial fluid uric acid and these cytokines were associated with the severity of knee OA rated on radiography and bone scintigraphy (32). IL-18, which is simultaneously activated by caspase-1 with IL-1β, was also associated with radiographic knee OA progression (32). With this finding, it is hypothesized that uric acid, either diffused into the joint from systemic circulation or released from dying chondrocytes, may form micro-particles with proteoglycan released from dying cells. These micro-particles may then trigger the innate immunity and NALP3 inflammasome pathway (32).

From all the *in vitro* studies that identify MSU crystals as an inflammasome activator, either lipopolysaccharide (LPS) or phorbol myristate acetate priming was required prior to the activation of the inflammasome by MSU crystals (33–35). Without priming, purified MSU crystals cannot induce IL-1β by themselves (33, 34), and induction rarely, if ever, occurs (35). Interestingly, in an *in vitro* study, Joosten et al. demonstrated the capacity of long chain free fatty acid (FFA) C18:0 to prime the innate immune system via toll-like receptor (TLR)-2, which resulted in activation of caspase-1 and release of IL-1β from peripheral blood leukocytes when exposed to MSU crystals (34). This is suggestive that systemic factors, such as FFA, may be important in interacting with MSU crystals to trigger the innate immune system, hence contributing to OA pathogenesis.

Lipopolysaccharide, a key pro-inflammatory product of the microbiome could be one such factor. Together with priming of innate immune system by LPS, damage-associated molecular patterns such as degraded cartilage fragments or MSU crystals may trigger TLR-4 leading to phagocytosis, inflammasome activation, and subsequent inflammation and joint damage (36). In a diet-induced obese rat (DIO) model that had greater body fat and more cartilage degradation compared to chow fed animals, Collins et al. showed that these DIO rats had a different gut microbiota composition and activity, higher plasma LPS and a distinct inflammatory profile in synovial fluid and serum (37). This suggests that a systemic influence due to diet, obesity and the microbiome may influence the pathogenesis of OA. In human, using the same cohort from the Prediction of Osteoarthritis Progression study, Huang et al. showed that both serum and synovial fluid LPS and the LPS binding protein were associated with an abundance of activated macrophages in the knee joint capsule and synovium, and severity of OA on radiography. Synovial fluid LPS was also positively associated with knee OA symptoms (38).

Uric acid could also be a predisposing factor that triggers the innate immune system. Crisan et al. pre-treated peripheral blood leukocytes with and without soluble uric acid for 24 h, and then stimulated these cells for 24 h with TLR-2 or -4 ligands in the presence and absence of MSU crystals (39). Uric acid pre-treatment was shown to enhance IL-1β, IL-6, and reduce IL-1 receptor antagonist production by peripheral blood leukocytes after exposure to MSU crystals (39). This study highlighted that chronic hyperuricemia may influence inflammatory responses by facilitating IL-1β production in peripheral blood leukocytes.

The necessity for a second factor to activate the MSU crystal-induced inflammation was reinforced by a recent *in vitro* study that evaluated the effect of IL-1β production and IL-1β mRNA expression by macrophages/monocytes on exposure to MSU crystals (40). Exposure of human macrophages/monocytes to MSU crystals alone did not induce the release of IL-1β, but required the presence of synovial fluid (supernatant and free of cells) from patients with inflammatory arthritis (40). On fractionation analysis, it was demonstrated that the MSU crystal co-stimulus was contained in the protein fraction but not in the lipidic fraction of synovial fluid (40). However, the protein from synovial fluid in this study acted as a co-stimulatory factor rather than a “primer” because pre-treatment of macrophages/monocytes with synovial fluid did not result in the production of IL-1β (40). It was noted that the synovial fluid from OA patients, in contrast to synovial fluid from inflammatory arthritis patients, did not affect the IL-1β production but slightly enhanced IL-8 secretion (40).

However, some studies suggested that cartilage degradation in OA may be independent of NLRP3 activation. In human OA joint explants, active IL-1β was predominantly produced by synovium rather than cartilage (41). Upon stimulation by inflammatory stress using LPS, IL-1α, and tumor necrosis factor (TNF)α, NLRP3-deficient mice showed similar cartilage degradation in the chondrocyte explants compared to wild-type mice (41). The authors also demonstrated that cartilage damage in these cartilage explants can be induced by direct mechanical forces rather than inflammatory stress. IL-1β and NLRP3 knock-out mice were not protected against cartilage damage induced by meniscectomy (42). Furthermore, in a collagenase-induced murine model of OA, IL-1α/β-deficient mice were not protected from cartilage loss and synovial inflammation (43). The conflicting data about the role of IL-1 in OA could be due to species differences and the method of OA induction. This also highlights the fact that OA is heterogeneous with numerous etiologies in different phenotypes, and the mechanistic pathways underlying inflammation in OA remains elusive.

**Figure 2:**
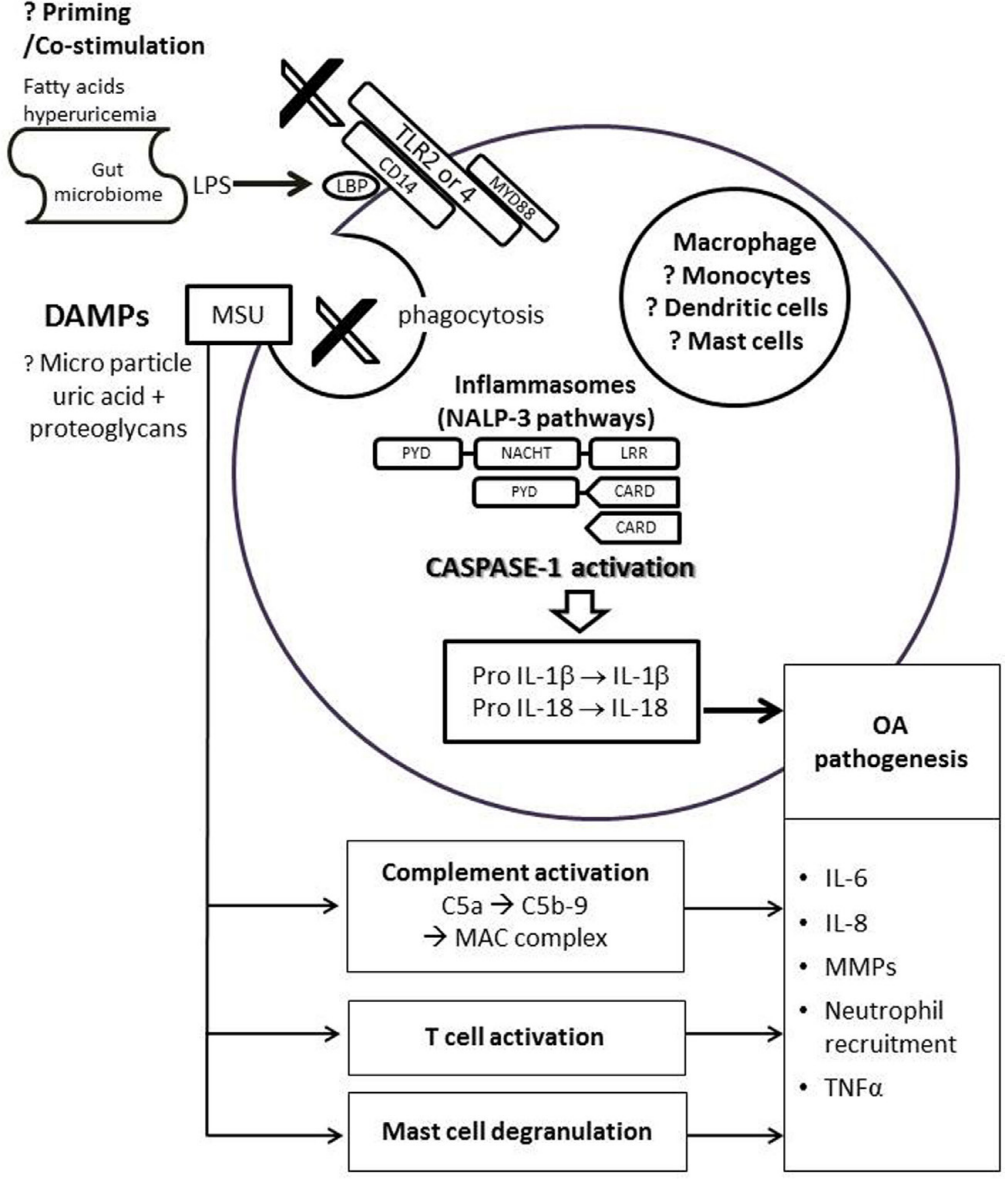
Possible link of uric acid in pathogenesis of osteoarthritis. DAMPs, such as monosodium urate (MSU) crystals or proteoglycans, bind to the TLR 2/4 and its co-receptor CD14 on immune cells. This triggers phagocytosis and assembly of NLRP3 inflammasome leads to activation of caspase-1, which in turn cleaves pro-IL-1β and pro-IL-18 to produce biologically active IL-1β and IL-18. IL-6, IL-8, TNF-α, and MMPs are also secreted by the immune cell, leading to neutrophil recruition and cartilage degradation. The activation of NLRP3 inflammasome pathway requires priming or co-stimulation of TLR2/4 by systemic factors such as fatty acids, hyperuricemia, or LPS from the gut microbiome that bind to the LBP on CD14. MSU is also linked to complement activation, direct T cell activation, and mast cell degranulation, all of which may be involved in the pathogenesis of OA. Abbreviations: CARD, caspase recruitment domain; DAMP, damage-associated molecular patterns; LPS, lipoproteinsaccaride; LBP, lipoproteinsaccaride-binding protein; IL, interleukin; LRR, leucine-rich repeat; MAC, membrane attack complex; NACHT, domain conserved in NAIP, CIITA, HET-E and TP1; NALP3, Nacht domain, leucine-rich repeat, and pyrin domain-containing protein 3; PYD, pyrin death domain; MMPs, matrix metalloproteinases; TLR, toll-like receptor; TNF, tumor necrosis factor.

Other immune cell types or pathways shown to be important for MSU-induced inflammation have been implicated in the pathogenesis of OA. MSU crystals can activate both the classical and alternative pathways of the complement system (44, 45), in particular MSU crystals cleave C5 directly on their surface and promote generation of C5a and C5b-9 involved in gouty inflammation (46, 47). Dysregulation of complement plays a crucial role in the pathogenesis of OA. Synovium from OA subjects had abnormally high expression and activation of complement (48). Mice deficient in C5, C6, or CD59a that cannot generate the terminal membrane attack complex (MAC) were resistant to the development of OA in three different mouse models of OA (48). MSU crystals can directly stimulate T cells (49), whereas altered T cell profiles (50), TNFα and other cytokines produced by T cells (51) have been implicated in the pathogenesis of OA. MSU crystals were shown to activate mast cells that play a central role in recruitment of polymorphs (52). Although the exact role of mast cell in OA is still unknown, activated mast cells are present in higher number in OA synovial tissues vs. rheumatoid arthritis, and a higher number of mast cells in synovial tissues was associated with greater degree of synovitis and structural damage (53). However, more details on how these pathways are linked in both conditions need further investigation (Figure 2).

### Therapeutic Implications

Despite the high prevalence and global impact of OA (3, 54), current treatments are palliative. Given its frequency, associated disability, and societal cost, subjects with OA are in urgent need of effective treatments that reduce the associated pain and symptoms, and slow its progression. Although many drugs have been approved for pain relief in OA (55), none have been approved for slowing of disease progression (56). The potential role of uric acid in pathogenesis of OA has given insight and raised the possibility of targeting hyperuricemia or the associated inflammatory pathways for OA trials. It is hypothesized that micro-particles formed by MSU crystals and proteoglycans from dying chondrocytes may induce a low-grade subclinical inflammation which worsens cartilage degradation and lead to knee OA progression (57). Colchicine, having multiple inhibitory effects on macrophages that include inhibition of the NALP3 inflammasome, inhibition of pore formation activated by purinergic receptors P2X7 and P2X2, and stimulation of dendritic cell maturation and antigen presentation, may be efficacious in relieving the symptom of knee OA (58). The pain and symptom relieving effects of colchicine for knee OA have been evaluated in several randomized controlled trials (Table 2) (59–62). Das et al. hypothesized that colchicine suppresses inflammation triggered by CPPD crystals and might have symptom modifying effects. The first clinical trial was conducted in 39 subjects with knee OA with clinical signs of inflammation, 74% had CPPD crystals in synovial fluid. Concomitant oral colchicine with intra-articular corticosteroid produced significantly greater symptomatic improvement and reduction of signs of knee inflammation compared to intra-articular corticosteroid alone (59). The same group conducted the second clinical trial in 36 ordinary subjects with moderately severe knee OA showed that regardless of presence or absence of inflammatory signs, colchicine added to nimesulide provide greater symptomatic improvement (defined by achieving a 30% reduction in a modified version of Western Ontario and McMaster Universities Arthritis Index, WOMAC) at 20 weeks than nimesulide alone (57.9% vs. 23.5%, *p* = 0.04) (60). Another study in Iran of 61 knee OA subjects without radiographic chondrocalcinosis demonstrated that colchicine added to usual treatment (analgesics, non-steroidal anti-inflammatory drugs, and physiotherapy) led to greater improvement in patient and physician global assessment at the end of three months compared to placebo (61).

**Table 2:** Summary of clinical trials of colchicine in knee OA.

Reference	Country, centers	Study design	Subject	n	Intervention	FU (weeks)	Outcome
Das et al. (59)	India, SC	RCT, DB, SC	OA knee with inflammation – Despite NSAIDs – All had IA steroid	39	Colchicine 0.5 mg bid vs. placebo	20	*Positive* Higher proportion achieved 30% improvement – Index knee VAS-pain (69 vs. 15%) – KGMC scores (74 vs. 45%)

Das et al. (60)	India, SC	RCT, DB, SC	Primary OA knee Addition to NSAID	36	Colchicine 0.5 mg bid vs. placebo	20	*Positive* Higher proportion achieved 30% improvement – Index knee VAS-pain (52.6 vs. 17.6%) – WOMAC (57.9 vs. 23.5%)

Aran et al. (61)	Iran, SC	RCT, DB	Primary OA knee Women, postmenopausal	61	Colchicine 0.5 mg bid vs. placebo	16	*Positive* – Less paracetamol consumption – Better patient global assessment – (11.14 ± 4.06 vs. 3.14 ± 2.18, *p* < 0.0001) – Better physician global assessment – (9.83 ± 3.8 vs. 3.72 ± 3.35, *p* < 0.0001)

Leung et al. (62) NCT02176460	Singapore, SC	RCT, DB	Primary OA knee	109	Colchicine 0.5 mg bid vs. placebo	16	*Negative* – No significant difference in proportion achieving primary end point (30% reduction in WOMAC) – (39 vs. 49%, *p* = 0.284) – Treatment significantly reduced serum hs-CRP and synovial fluid CTXI

*OA, osteoarthritis; RCT, randomized controlled trial; DB, double-blinded; SC, single center; NSAIDs, non-steroidal anti-inflammatory drugs; IA, intra-articular; vs., versus; bid, twice daily; VAS, visual analog scale; KGMC, total King George’s Medical College (KGMC) scale; WOMAC, total Western Ontario and McMaster University Osteoarthritis index; hs-CRP, high sensitive C-reactive protein; CTXI, cross-linked C-telopeptide of type I collagen*.

Our group recently reported the results of our randomized double-blinded, placebo-controlled trial with a larger sample size (NCT02176460) (57, 62). We randomized 109 subjects with knee OA and moderate pain to receive 0.5 mg oral colchicine or placebo twice daily for 16 weeks. Subjects with a history of gout or prodagra were excluded. Synovial fluid was examined under polarized microscopy to rule out the presences of MSU crystals before commencement. At the study end, there was no significant difference between subjects in the colchicine and placebo arms who achieved the primary end point, defined by a 30% reduction in WOMAC total {39% vs. 49%, *p* = 0.284, odds ratio = 0.66 [95% confidence interval (0.31–1.41)]}. A high placebo response was noted. No strong evidence of treatment differences was identified on secondary endpoints, including pain scores, physical function, the OARSI-OMERACT response, and quality of life. No significant differences were noted within the treatment arms in subgroup analysis when stratified by age, gender, uric acid, radiographic severity and high vs. low serum high sensitive C-reactive protein (hs-CRP). Colchicine was well tolerated with good safety profile. The change in synovial fluid CD14, a marker of activated macrophage, correlated significantly with change in synovial fluid IL-18 in the colchicine but not the placebo arm. This is consistent with our hypothesis that colchicine suppresses pro-inflammatory cytokine production via suppression of activated macrophages. Interesting finding in this study was that colchicine treatment significantly reduced mean serum hs-CRP (*p* = 0.008) and synovial fluid CTXI (*p* = 0.002). A high serum CTXI predicted clinically relevant (knee structural and symptom) OA progression over 48 months from the Foundation for the National Institutes of Health study (63), while a high serum MMP-degraded CRP, or CRP-M predicted incident radiographic knee OA over three years (64). These results raise the intriguing possibility that colchicine may have slow-acting disease modification potential in OA. Colchicine has demonstrated potential cardiovascular benefits with in recent clinical trials (65, 66). Given the safety profile of standard clinical doses of colchicine in our study and others, long-term colchicine could warrant further consideration for evaluation of structural disease-modifying effects using sensitive measures such as magnetic resonance imaging in OA. In particular, a longer term trial would be needed to determine if the biological indications of a possible effect are reflected over time in patient-reported benefits.

## Conclusion

There is no conclusive causal relationship between uric acid, gout, and OA thus far. Emerging evidence from epidemiologic studies, however, supports an association between gout and OA, after controlling for BMI, the most significant confounding factor. New understanding on the role of inflammation in both crystal-induced arthritis and OA has given insights into a possible shared pathogenesis pathway. These are good justifications for further studies to understand the link between the two common conditions and evaluate the possibility of repurposing traditional gout medication for use in OA.

## Author Contributions

YYL conceptualized and designed the study; both authors reviewed the literature and acquired the data; both authors drafted the manuscript and approved the final version of manuscript. YYL took responsibility for the integrity of the work as a whole.

## Conflict of Interest Statement

The authors declare that the research was conducted in the absence of any commercial or financial relationships that could be construed as a potential conflict of interest.

## Abbreviations

ADAMTS, a disintegrin and metalloproteinase with thrombospondin motifs; BMI, body mass index; CTXI, cross linked C-telopeptide of type I collagen; hs-CRP, high-sensitive C-reactive protein; CRP-M, matrix metalloproteinase-degraded C-reactive protein; FFA, free fatty acid; HR, hazard ratio; IL, interleukin; LPS, lipopolysaccharide; NALP3, nacht domain, leucine-rich repeat, and pyrin domain-containing protein 3; MSU, monosodium uric acid crystal; OA, osteoarthritis; OARSI-OMERACT response, responder criteria by the Osteoarthritis Research Society International and Outcome measures in Rheumatology; OR, odds ratio; TNFα, tumor necrosis factor α; UK, United Kingdom.
